# MiR-122-5p promotes peritoneal fibrosis in a rat model of peritoneal dialysis by targeting Smad5 to activate Wnt/β-catenin pathway

**DOI:** 10.1080/0886022X.2022.2030360

**Published:** 2022-02-16

**Authors:** Yirong Liu, Zhihong Ma, Zhenxing Huang, Dongmei Zou, Junbin Li, Ping Feng

**Affiliations:** a Department of Nephrology, Xining No.1 People's Hospital, Xining, PR China; b Department of Endocrinology, Xining No.1 People's Hospital, Xining, PR China

**Keywords:** Peritoneal fibrosis, epithelial–mesenchymal transition (EMT), MiR-122-5p, Smad5, Wnt/β-catenin

## Abstract

Peritoneal fibrosis (PF) is the main reason leading to declining efficiency and ultrafiltration failure of peritoneum, which restricts the application of peritoneal dialysis (PD). We aimed to investigate the effects and mechanisms of miR-122-5p on the PF. Sprague-Dawley (SD) rats were infused with glucose-based standard PD fluid to establish PF model. HE staining was performed to evaluate the extent of PF. Real-time fluorescence quantitative polymerase chain reaction (RT-qPCR) and fluorescence *in situ* hybridization (FISH) were performed to measure the expression level of miR-122-5p. Western blot was used to test the expression of transforming growth factor (TGF)-β1, platelet-derived growth factor (PDGF)-A, Fibronectin 1 (FN1), extracellular matrix protein 1 (ECM1), Smad5, α-smooth muscle actin (SMA), collagen type 1(COL-1), Vimentin, E-Cadherin, Wnt1, β-catenin, p-β-catenin, c-Myc, c-Jun, and Cyclin D1. Immunohistochemistry (IHC) staining was used to detect type I collagen alpha 1 (Col1α1), α-SMA, and E-Cadherin expression. We found PF was glucose concentration-dependently enhanced in peritoneum of PD rat. The PD rats showed increased miR-122-5p and decreased Smad5 expression. MiR-122-5p silencing improved PF and epithelial–mesenchymal transition (EMT) process in PD rats. MiR-122-5p silencing attenuated the activity of the Wnt/β-catenin signaling pathway. Importantly, dual-luciferase reporter assay showed Smad5 was a target gene of miR-122-5p. Smad5 overexpression significantly reversed the increases of PF and EMT progression induced by miR-122-5p overexpression. Moreover, miR-122-5p mimic activated Wnt/β-catenin activity, which was blocked by Smad5 overexpression. Overall, present results demonstrated that miR-122-5p overexpression showed a deterioration effect on PD-related PF by targeting Smad5 to activate Wnt/β-catenin pathway.

## 1. Introduction

Peritoneal dialysis (PD) is the main alternative treatment for end-stage renal disease (ESRD), and approximately 11% of ESRD patients worldwide require PD [1,2]. PD mainly relies on peritoneum as a biological semi-permeable membrane for ultrafiltration and solute diffusion [3]. However, long-term PD treatment will lead to peritoneal fibrosis (PF), resulting in the loss of structure and function of the peritoneum. The PF ultimately leads to failure of peritoneal ultrafiltration which is the main reason of dropping out of treatment for PD patients [4]. Thus, prevention and delay of PF were the key to ensuring long-term success for PD patients. The studies have found that epithelial–mesenchymal transition (EMT) is the triggering and initiating factor in the process of PF [5,6]. EMT can lead to the transformation of peritoneal mesothelial cells into fibroblasts and produce a large amount of extracellular matrix (ECM), which is a key process of peritoneal function degradation. In recent years, although many related preclinical studies have made significant breakthroughs, there is still a lack of therapeutic targets to relieve and intervene in PF.

MicroRNAs (miRNAs) are 21–25 nucleotide non-coding RNA molecules [7]. MiRNAs have been identified only 1–3% of the human genome sequence, but regulate in 1/3 of the gene progression usually by binding to 3'-untranslated regions (3'-UTRs) of mRNA, which are involved in multiple cell activities, including cell differentiation, proliferation, apoptosis, and so on. Many studies have shown that the disorders of miRNAs expression are associated with many diseases, and play a key role in development progress of EMT [8–10]. Importantly, microarray analysis proved that various miRNAs including miR-122-5p were differentially expressed in the serum and drained dialysate of PD patients [11]. Meanwhile, in the rat model of PF, the expression of miR-122 in peritoneum tissue was significantly up-regulate in the hypertonic dialysate group compared with the control and the saline groups, which may play an important role in pathogenesis of PF [12]. Mechanically, miR-302c [13], miR-21, and miR-589 [14] modulated PD-associated fibrosis or correlated with peritoneal transport characteristics by regulate the expression of multiple target genes. MiR-122-5p is believed to be involved in regulating tumor cell growth, migration, metastasis, and EMT [15,16], but the effect of miR-122-5p on PF is unknown. Therefore, this study investigated the expression and effect of miR-122-5p in PF by mediating the Wnt/β-catenin signaling pathway *via* the Smad5 gene.

## 2. Methods

### 2.1. Cell culture and cell transfection

Human peritoneal mesothelial cells HMrSV5 were acquired from BeNa Culture Collection Company (Beijing, China). All of the cells were cultured in DMEM/F12 culture medium (Gibco, Carlsbad, CA) supplemented with 10% fetal bovine serum (FBS; Gibco-BRL Life Technologies, Paisley, UK) and antibiotics (100 U/mL penicillin and 100 μg/mL streptomycin; Sigma-Aldrich Co., St. Louis, MO) at 37 °C with 5% CO_2_ in a humidified incubator. HMrSV5 cells (1.0 × 10^5^ cells/well) were seeded into 6-well plates 24 h prior to transfection/treatment. Lipofectamine 2000 transfection reagent was used for the transient transfection of NC inhibitor, miR-122-5p inhibitor, NC mimic, or miR-122-5p mimic (5′-UGG AGU GUG ACA AUG GUG UUU G-3′) according to the manufacturer's instructions (RiboBio, Guangzhou, China).

### 2.2. Construction of a rat model of PF

A total of 64 Sprague-Dawley (SD) rats (180–200 g) were purchased from Chengdu Dossy Experimental Animals CO., LTD.(Chengdu, Sichuan; SCXY (Chuan) 2020-034). All experiments were approved according to the Ethics Committee of Xining First People's Hospital (2020-LLPJ-22). A total of 24 rats were used in the first part of the experiment. Four rats died during the experiment (mortality rate 16.7%), including two rats in 4.25%-Glu group, one rat in 3.25%-Glu group, and one rat in 2.25%-Glu group, probably due to low activity and poor diet. Different glucose concentrations of peritoneal dialysate (4.25%/3.25%/2.25%, 15 mL) were intraperitoneally injected into the rats for eight consecutive weeks in the PF model group to establish the experimental model. Equal volumes of saline were administrated to the rats in the control group *via* intraperitoneal injections as the control. The number of rats used for follow-up experiments was: *n* = 6 (Control group), *n* = 4 (4.25%-Glu group), *n* = 5 (3.25%-Glu group), and *n* = 5 (PD + 2.25%-Glu group). Moreover, 40 rats were used in the second part of the experiment. We then used random numbers to divide these rats into five groups. Rats in the control group received daily intraperitoneal injections of saline solution (0.9% NaCl) for 4 weeks. Rats in the PD group received 4.25% standard PD fluid. Rats in the lentiviral shuttle plasmid pCD513B-Smad5 group injected intraperitoeally with lentiviral shuttle plasmid pCD513B-Smad5. Rats in the miR-122-5p mimic group injected intraperitoeally with miR-122-5p mimic. Seven rats died during the experiment (mortality rate 17.5%), including one rat in PD group, two rats in PD + pCD513B-Smad5 group, two rats in PD + miR-122-5p mimic group, and two rats in PD + pCD513B-Smad5 + miR-122-5p mimic group. The number of rats used for follow-up experiments was: *n* = 8 (Control group), *n* = 7 (PD group), *n* = 6 (PD + pCD513B-Smad5 group), *n* = 6 (PD + miR-122-5p mimic group), and *n* = 6 (PD + pCD513B-Smad5 + miR-122-5p mimic group). Finally, 18 rats were used in the third part of the experiment. We used random numbers to divide these rats into three groups. NC inhibitor/miR-122-5p inhibitor was injected intraperitoeally into rats. Two rats died during the experiment (mortality rate 11.1%), including one rat in PD group and one rat in PD + miR-122-5p inhibitor group. The number of rats used for follow-up experiments was: *n* = 6 (Control group), *n* = 5 (PD + NC inhibitor group), *n* = 5 (PD + miR-122-5p inhibitor). All rats were housed under natural lighting with free access to food and water. After the experiment, the rats were anesthetized using intraperitoneal pentobarbital (50 mg/kg). Then the animals were euthanized by aortic exsanguinations, the abdomen was opened by a midline incision, and the entire anterior abdominal wall was removed for further examination. The experimental procedures were approved by animal ethical committee of Xining First People’s Hospital. The experiments complied with the ARRIVE guidelines and carried out in accordance with the U.K. Animals (Scientific Procedures) Act, 1986 and associated guidelines, EU Directive 2010/63/EU for animal experiments. The animal care was in accordance with National Institutes of Health guide.

### 2.3. Hematoxylin and eosin (H&E) staining

The superior lobe of the parietal peritoneum tissue was kept in 10% neutral formaldehyde for histology. Next, samples of peritoneum tissues were embedded in paraffin, sectioned (4 μm thickness), and mounted on to slides. According to the instructions, the sections were soaked in xylene, gradient concentrations of Hematoxylin and Eosin (H&E), respectively. After that, they were dried and tissue lesions were examined under a light microscope.

### 2.4. Real-time fluorescence quantitative polymerase chain reaction (RT-qPCR)

The different groups of peritoneum tissues or cells were harvested and their total RNAs were isolated by using TRIzol^®^ reagent (Thermo Fisher, Waltham, MA). The cDNA was obtained using reverse transcription kit (Invitrogen, Carlsbad, CA). The relative levels of target gene RNA transcripts were determined by real-time fluorescence quantitative polymerase chain reaction (RT-qPCR) by using a SYBR Pemix Ex Taq kit (Bao Biological Engineering, Dalian, China). The reverse transcriptional reaction condition was as follows: 95 °C for 30 s, 40 cycles of 95 °C for 5 s, and 60 °C for 30 s. The U6 small nuclear RNA was used to the internal control. The relative gene expression level was determined using the 2−^△△Ct^ method on ABI software, Foster City, CA.

### 2.5. Western blot analysis

Total protein was extracted from peritoneal tissues or cells using RIPA buffer (cat. no. P0013C; Beyotime Institute of Biotechnology, Hangzhou, China). Protein concentrations were determined using a BCA assay kit (CoWin Biosciences, Cambridge, MA). Then the proteins were separated by 10% SDS polyacrylamide gel electrophoresis and then transferred to nitrocellulose membranes. The membranes were blocked with 5% nonfat milk and incubated with corresponding protein antibodies or a rabbit anti-β-actin monoclonal antibody. Then, the membranes were subsequently incubated with a HRP Goat anti-Rabbit IgG (Abcam, Cambridge, UK; cat. no. ab6721). The net optical density (OD) was analyzed with the gel Image processing system (Image-pro Plus 6.0). Primary antibodies used were as follows: Transforming growth factor (TGF)-β1 (Abcam; cat. no. ab215715; 1/1000), Wnt1 (Abcam; cat. no. ab63934; 1/500), β-catenin (Abcam; cat. no. ab32572; 1/5000), phospho-β-Catenin Ser675 (Cell Signaling, Danvers, MA; cat. no. 9567; 1/1000), Fibronectin 1 (FN1; Cell Signaling; cat. no.2413; 1/5000), platelet-derived growth factor (PDGF)-A (Abcam; cat. no. ab203911; 1/1000), Smad5 (Abcam; cat. no. ab40771; 1/1000), ECM protein 1 (Abcam; cat. no. ab253185; 1/1000), α-smooth muscle actin (SMA; Cell Signaling; cat. no. 19245; 1/1000), collagen type 1(COL-1)-1 (Abcam; cat. no. ab6308; 1/1000), Vimentin (Abcam; cat. no. ab92547; 1/1000), E-Cadherin (Abcam; cat. no. ab40772; 1/10000), c-Jun (Abcam; cat. no. ab 40766; 1/1000), c-Myc (Abcam; cat. no. ab 32072; 1/1000), Cyclin D1 (Abcam; cat. no. ab 16663; 1/50), and β-actin (BM0627, Boster, Wuhan, China; 1/1000). The bands were visualized using the ECL system (Affinity Biosciences, Cincinnati, OH) and β-actin was used as an internal control.

### 2.6. Dual-luciferase reporter assay

The wild type (wt) or mutated (mut) of Smad5 3'-UTR, containing the binding sites of miR-122-5p were inserted into psiCHECK-2 vector (Promega, Madison, WI). Then, the luciferase reporter vectors and miR-122-5p mimic or NC mimic were co-transfected into HEK-293T cells (4 × 10^4^ cells/well) with the Lipofectamine 2000 (Invitrogen, Carlsbad, CA) following the manufacturer’s instructions. After transfection for 24 h, the relative luciferase activities were measured by a luciferase reporter assay system.

### 2.7. Immunohistochemical (IHC) staining

Paraffin sections were dewaxed with xylene and dehydrated with gradient alcohol. Antigen repair was performed with 0.01 mol/L citrate buffer solution. 3% hydrogen peroxide was used to block endogenous peroxidase. After cooling naturally at room temperature, the paraffin sections were washed with 0.1 mol/L PBS and blocked with 5% BSA. Then, the paraffin sections were incubated overnight with rabbit anti-type I collagen (Col1α1, 1:100). Finally, the paraffin sections were subsequently incubated with HRP-labeled goat anti-rabbit secondary antibody. The proteins were detected at room temperature using a DAB immunohistochemistry (IHC) color development kit (Boshide Biological, Wuhan, China). ImagePro Plus image analysis software was used to analyze the OD value of the slice.

### 2.8. Fluorescence *in situ* hybridization (FISH)

The expression level of miR-122-5p in peritoneal tissues was evaluated by fluorescence *in situ* hybridization (FISH) using an alexa Fluor 488-labeled miR-122-5p probe (RiboBio, Guangzhou, China). The probe signals were determined with the FISH Kit (BersinBio, Guangzhou, China) according to the manufacturer’s guidelines. Images were acquired using a fluorescence microscope (BX53, Olympus, Tokyo, Japan). The Image-J image analysis system was used to measure the fluorescence intensity (Integrated Density, IntDen) and area of all collected images, and calculate the average fluorescence intensity of each image.

### 2.9. Statistical analysis

The data were represented as means ± standard deviation (SD). Statistical analysis was performed with the SPSS software version 19.0 (SPSS Inc., Chicago, IL). One-way analysis of variance (ANOVA) followed by Tukey’s *post hoc* test and Student’s unpaired t-test were used for comparison between groups. *p* Value < .05 was considered statistically significant.

## 3. Results

### 3.1. Observation of the peritoneal tissue staining

**Figure 1.**
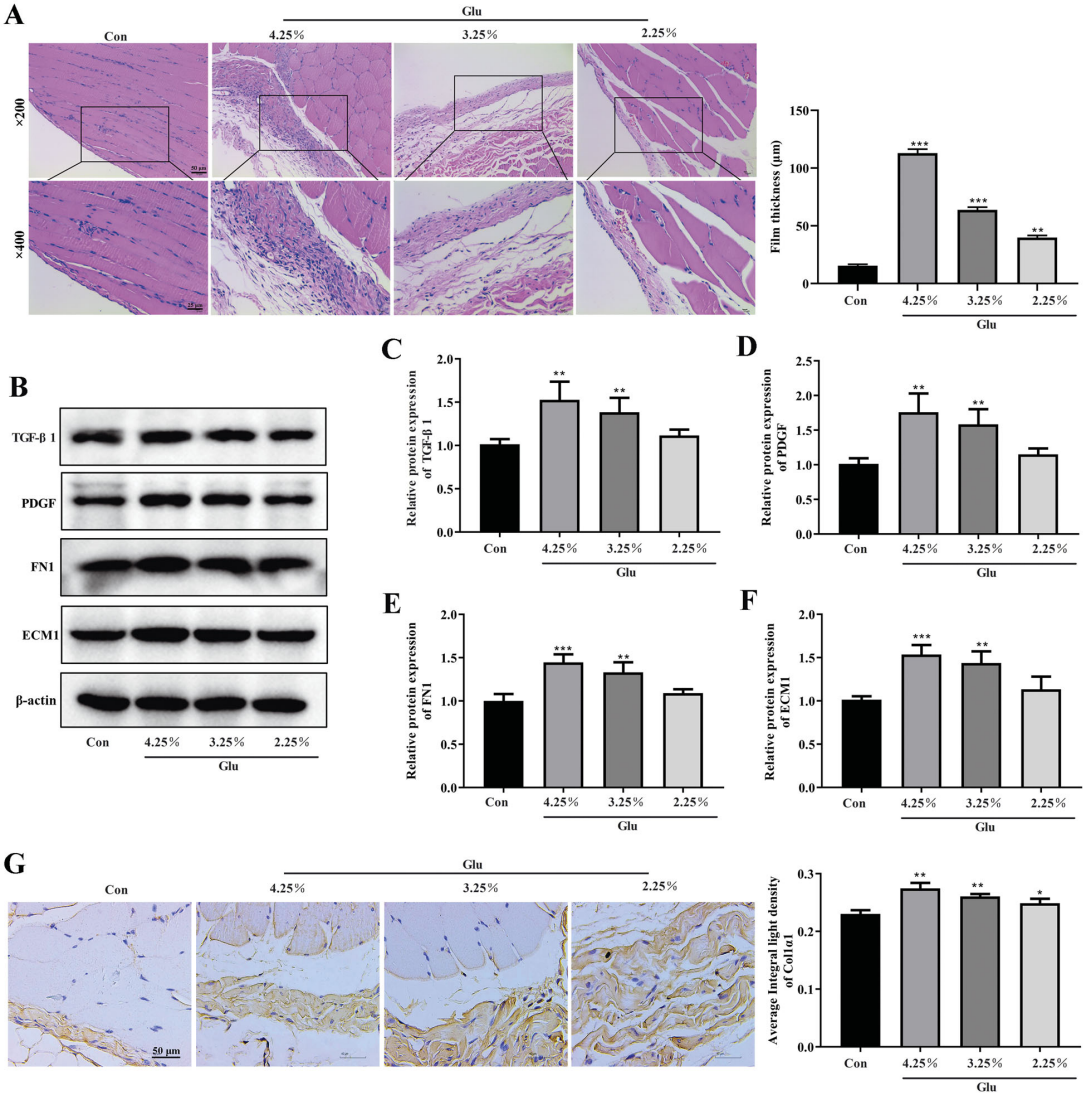
Observation of the peritoneal tissue staining. (A) Hematoxylin-eosin (HE) staining of the peritoneal membrane. (scale bar = 50 and 25 μm) (B–F) Western blot shows the expression of TGF-β1, PDGF, FN1, and ECM1 in peritoneum of different groups. (G and H) Immunohistochemical of Col1α1 expression (scale bar = 50 μm). *p<.05, **p<.01, ***p<.001 *versus* the control (Con) group. *n* = 6 (Co group), *n* = 4 (4.25%-Glu group), *n* = 5 (3.25%-Glu group), and *n* = 5 (PD + 2.25%-Glu group).

First, the rat PF model was successfully established in our study. As shown in Figure 1(A), the H&E staining results demonstrated that the peritoneum of normal rats was integrated and continuous with a thin dense and smooth morphology, covered with a layer of mesothelial cells. However, the peritoneum in fibrosis rats treated with peritoneal dialysate containing different concentrations of glucose exhibited a disorder structure, with significantly augmented thickness, cylindrical or circular and exfoliated mesothelial cells, as well as inflammatory cell infiltration with edema (Figure 1(A)). In addition, we detected the changes in related indicators to verify the occurrence of PF. The expression of TGF-β1, PDGF, FN1, and ECM1 was concentration-dependently enhanced in peritoneum of PD rat (Figure 1(B–F)). It is well known that the accumulation of type I collagen is characteristic of the fibrotic process. Our immunohistochemical results showed that the expression of alpha1 subunit of Col1α1 was increased with the increase of the concentration of PD solution in peritoneum of PD rat (Figure 1(G)).

### 3.2. The expression of miR-122-5p was up-regulated in rat model of PD

**Figure 2.**
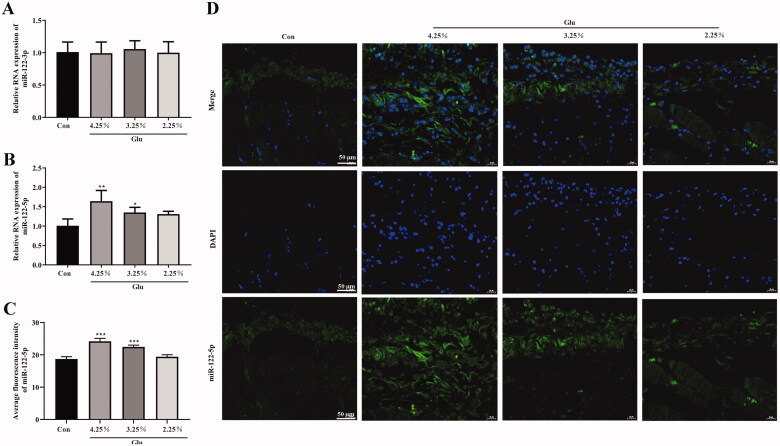
The expression of miR-122-5p was up-regulated in rat model of PD. (A and B) The miR-122-3pand miR-122-5p expression level in peritoneum was analyzed by RT-qPCR. (C and D) fluorescence *in situ* hybridization was used to test the intracellular localization of miR-122-5p (scale bar = 50 μm). *p<.05, **p<.01, ***p<.001 *versus* the control (Con) group. *n* = 6 (Co group), *n* = 4 (4.25%-Glu group), *n* = 5 (3.25%-Glu group), and *n* = 5 (PD + 2.25%-Glu group).

We then tested the expression of miR-122-3p and miR-122-5p in peritoneal tissue. The results of RT-qPCR analysis showed that there was no difference in the expression of miR-122-3p between the groups (Figure 2(A)). The expression of miR-122-5p was significantly weak in the peritoneal tissue cells of the normal rats, while that of PF rats was rather increased dependently with glucose concentration (Figure 2(B)). Furthermore, FISH showed that miR-122-5p was highly expressed in peritoneal mesothelial cells and vascular endothelial cells of peritoneal tissue in a glucose-concentration-dependent manner compared with the control group (Figure 2(C,D).

### 3.3. Mir-122-5p down-regulating inhibited peritoneal fibrosis and EMT progression

**Figure 3.**
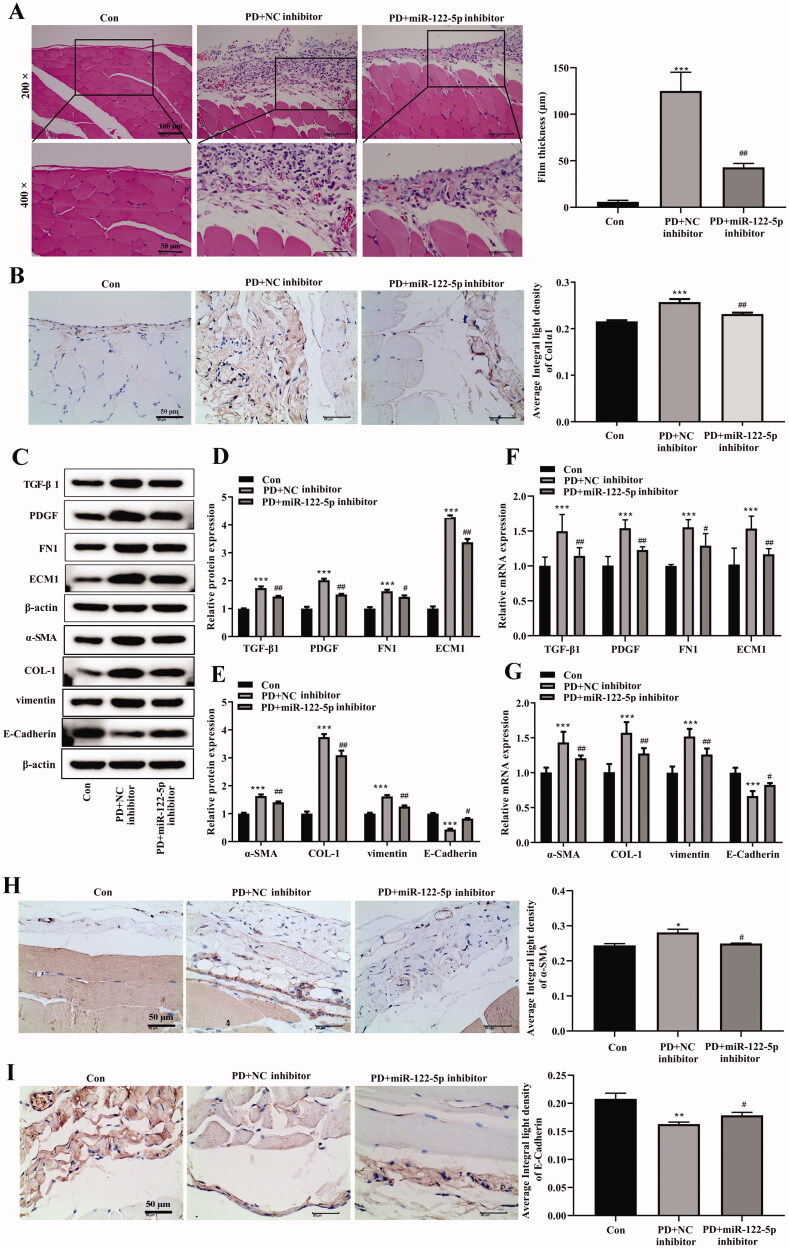
MiR-122-5p down-regulating inhibited peritoneal fibrosis and EMT progression. (A) Hematoxylin-eosin (HE) staining of the peritoneal membrane. (B) Immunohistochemical of Col1α1 expression. (C–G) Western blot shows the expression of TGF-β1, PDGF, FN1, ECM1, α-SMA, COL-1, COL-1, vimentin, and E-Cadherin in peritoneum of different groups. (H) Immunohistochemical of α-SMA expression. (I) Immunohistochemical of E-Cadherin expression. *p<.05, **p<.01, ***p<.001 *versus* the control (Con) group. ^#^*p*<.05, ^##^*p*<.01 *versus* the PD + NC inhibitor group. *n* = 6 (Con group), *n* = 5 (PD + NC inhibitor group), *n* = 5 (PD + miR-122-5p inhibitor group).

Then, we explored the mechanism of miR-122-5p on PF and EMT progression by down-regulating miR-122-5p expression using miR-122-5p inhibitor. MiR-122-5p inhibitor reduced peritoneum fibrosis compared with the PD group (Figure 3(A)). IHC staining indicated that miR-122-5p silencing suppressed the expression of Col1α1 in peritoneum and induced the expression of TGF-β1, PDGF, FN1, and ECM1 in the PD group (Figure 3(C,D,F)). It was reported that EMT was one of the important mechanisms during the pathogenesis of PF. Thus, we evaluated the expression of epithelial and mesenchymal marker proteins in peritoneum by Western blot or IHC. Compared with the control group, E-cadherin expression was significantly decreased, as well as the expression of α-SMA, COL-1, and vimentin was increased in peritoneum of the PD group (Figure 3(C,E,G–I)). Intriguingly, miR-122-5p inhibitor inhibited α-SMA, COL-1, and vimentin expression and promoted E-cadherin expression in peritoneum (Figure 3(C,E,G–I)).

### 3.4. Mir-122-5p down-regulating inhibited the activity of canonical Wnt/β-catenin signaling pathway

**Figure 4.**
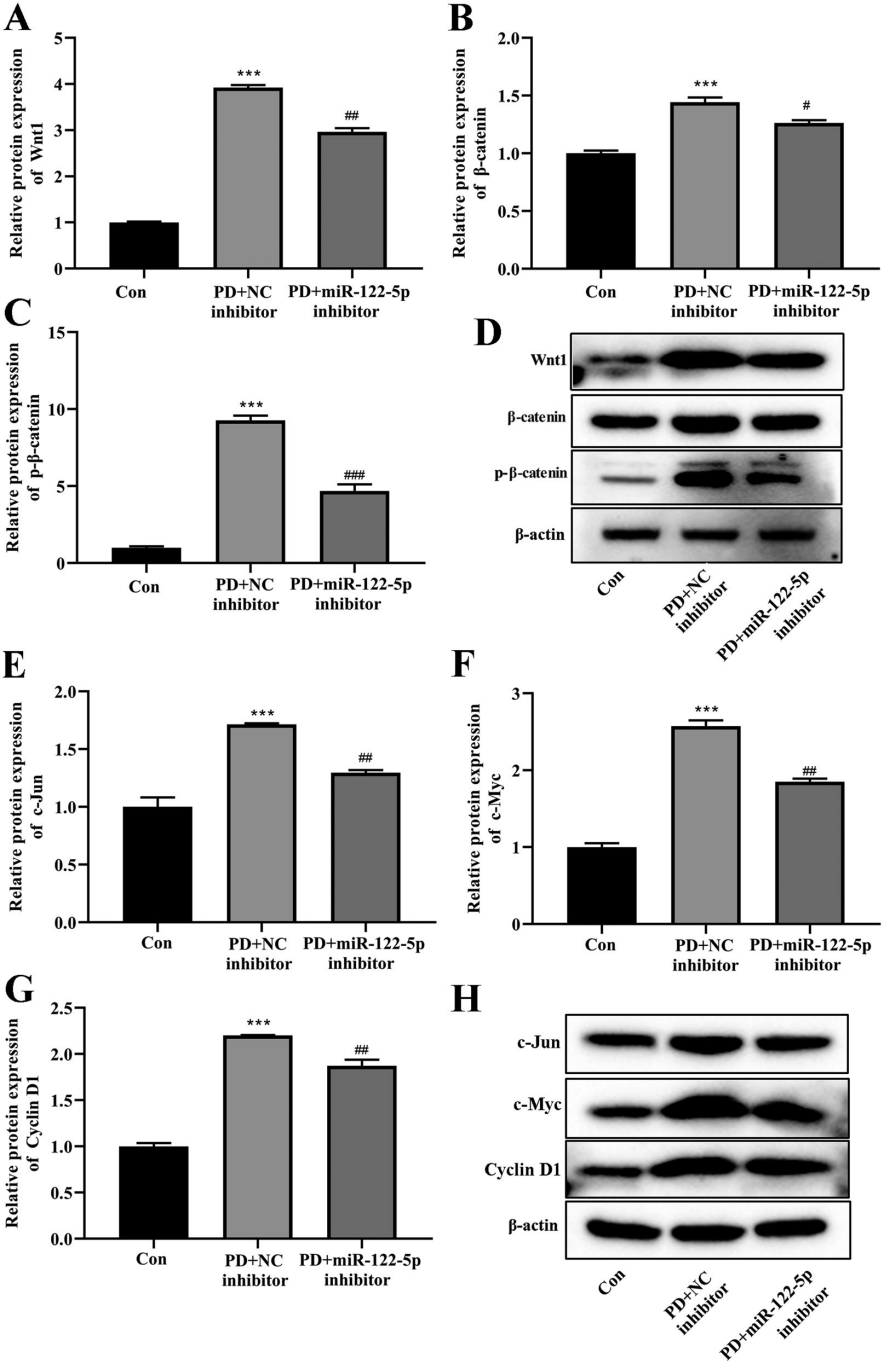
MiR-122-5p down-regulating inhibited the activity of canonical Wnt/β-catenin signaling pathway. Western blot showed the expression of Wnt1, β-catenin, p-β-catenin, c-Jun, c-Myc, and Cyclin D1 in peritoneum of different groups. ***p< .001 *versus* the control (Con) group. ^#^*p*<.05, ^##^*p*<.01, ^###^*p*<.001 *versus* the PD + NC inhibitor group. *n* = 6 (Con group), *n* = 5 (PD + NC inhibitor group), *n* = 5 (PD + miR-122-5p inhibitor group).

Wnt/β-catenin signaling pathway plays an important role in the occurrence and development of various fibrotic diseases, including PF. We further found that the expression of canonical Wnt/β-catenin signaling-related proteins Wnt1 and β-catenin was enhanced in the peritoneal tissue of PD rats (Figure 4(A,B,D)). In addition, the phosphorylation level of β-catenin was also increased compared with the control group (Figure 4(C,D)). MiR-122-5p inhibitor obviously reversed these changes. Meanwhile, PD induced increases of Wnt/β-catenin signaling target genes C-Jun, c-Myc, and Cyclin D1 were also hindered by miR-122-5p silencing in peritoneum (Figure 4(E–H)).

### 3.5. Smad5 was a potential target of miR-122-5p

**Figure 5.**
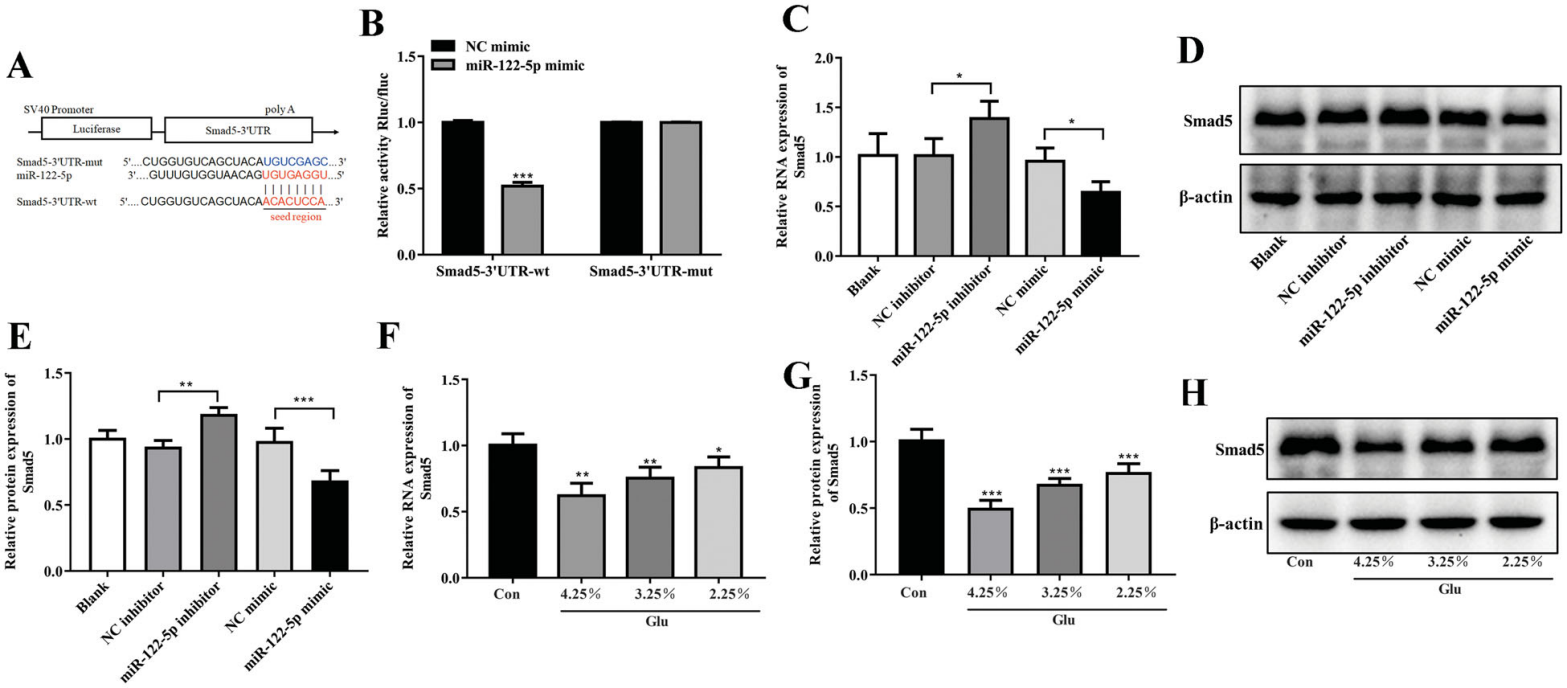
Targeting relationships between miR-122-5p and Smad5. (A) Prediction of the binding site between miR-122-5p and Smad5 by TargetScan. (B) Luciferase activity of HEK-293T cells transfected with a luciferase reporter containing the wild-type or mutated 3'-UTR of Smad5. Then, cells were transfected with miR-122-5p mimic or NC mimic, and the normalized levels of luciferase activity were determined. *n* = 3. (C–E)) The RNA and protein expression of Smad5 in HMrSV5 was tested by RT-qPCR and Western blot, respectively. *n* = 3. (F–H) The RNA and protein expression of Smad5 in peritoneum was tested by RT-qPCR and Western blot, respectively. *n* = 6 (Co group), *n* = 4 (4.25%-Glu group), *n* = 5 (3.25%-Glu group), and *n* = 5 (PD + 2.25%-Glu group). *p<.05, **p<.01, ***p<.001 *versus* NC mimic/NC inhibitor/the control (Con) group.

The target gene of miR-122-5p was predicted by bioinformatics software starBase version 2.0 (https://starbase.sysu.edu.cn/starbase2/index.php), of which the results suggested that there was a potential binding site of miR-122-5p on the 3'-UTR of Smad5 (Figure 5(A)). Meanwhile, dual luciferase reporter assay also proved that after transfected with psiCHECK-2-Smad5-wt plasmids, the luciferase activity of HEK-293T cells was significantly decreased in miR-122-5p mimic group compared with NC group (Figure 5(B)). Furthermore, the results of RT-qPCR and Western blot indicated that the expression of Smad5 was negatively correlated to that of miR-122-5p in HMrSV5 cells (Figure 5(C–E)). These results suggested that miR-122-5p could directly bind to 3'-UTR of Smad5 and inhibit the transcriptional activity of Smad5. Additionally, according to the results of the Western blot and RT-qPCR analysis, PF dose-dependently decreased Smad5 expression (Figure 5(F–H)).

### 3.6. Mir-122-5p aggravated peritoneal fibrosis and EMT progression by targeting Smad5 *in vivo*

**Figure 6.**
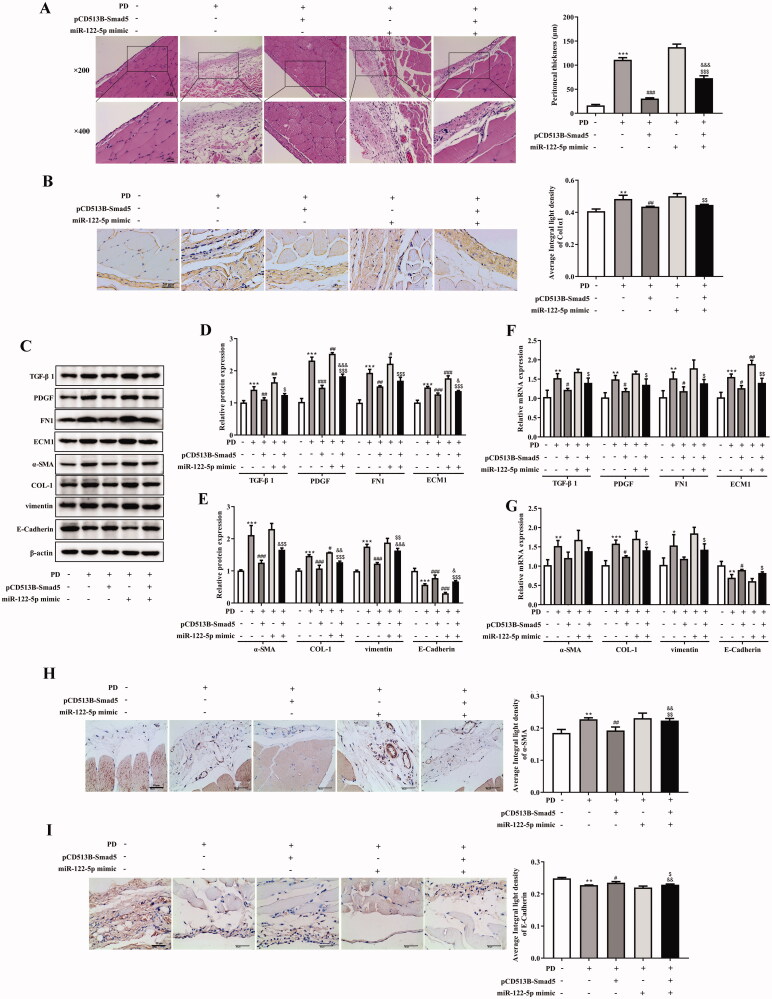
MiR-122-5p aggravated peritoneal fibrosis and EMT progression by targeting Smad5 *in vivo*. (A) Hematoxylin-eosin (HE) staining of the peritoneal membrane. (B) Immunohistochemical of Col1α1 expression. (C-G) Western blot shows the expression of TGF-β1, PDGF, FN1, ECM1, α-SMA, COL-1, COL-1, vimentin, and E-Cadherin in peritoneum of different groups. (H) Immunohistochemical of α-SMA expression. (I) Immunohistochemical of E-Cadherin expression.**p<.01, ***p<.001 *versus* the control (Con) group. ^#^*p*<.05, ^##^*p*<.01, ^###^*p*<.001 *versus* the PD group. ^&^*p*<.05, ^&&^*p*<.01, ^&&&^*p*<.001 *versus* pCD513B-Smad5 treatment group. ^$^*p*<.05, ^$$^*p*<.01, ^$$$^*p*<.001 *versus* miR-122-5p mimic treatment group. *n* = 8 (Con group), *n* = 7 (PD group), *n* = 6 (PD + pCD513B-Smad5 group), *n* = 6 (PD + miR-122-5p mimic group), and *n* = 6 (PD + pCD513B-Smad5 + miR-122-5p mimic group).

We further demonstrated the effects and underlying mechanisms of miR-122-5p on PF. H&E staining indicated that peritoneum of the PD group showed different degrees of fibrosis. MiR-122-5p treatment aggravated fibrosis of peritoneal tissues which could block by pCD513B-Smad5 treatment (Figure 6(A)). As shown in Figure 6(B), the expression of Col1α1 in the peritoneum was significantly higher in the PD group than in the control group, however, Smad5 overexpression treatment reduced this level and miR-122-5p mimic treatment-induced this level. Meanwhile, Smad5 overexpression blocked the expression of Col1α1 which induced by miR-122-5p mimic in Smad5 overexpression and miR-122-5p mimic co-treatment group (Figure 6(B)). More importantly, the expression of TGF-β1, PDGF, FN1, and ECM1 was increased by miR-122-5p mimic compared with PD group (Figure 6(C–E)). And Smad5 overexpression dramatically reversed these increases (Figure 6(C–E)). Furthermore, we found such protective effect of Smad5 as associated with the blockade of EMT by preventing the loss of the epithelial marker E-cadherin, and the gain of the mesenchymal marker α-SMA, COL-1, and vimentin (Figure 6(C,FG–I)).

### 3.7. Overexpression of miR-122-5p enhanced PD-induced activation of canonical Wnt/β-catenin signaling pathway by targeting Smad5

**Figure 7.**
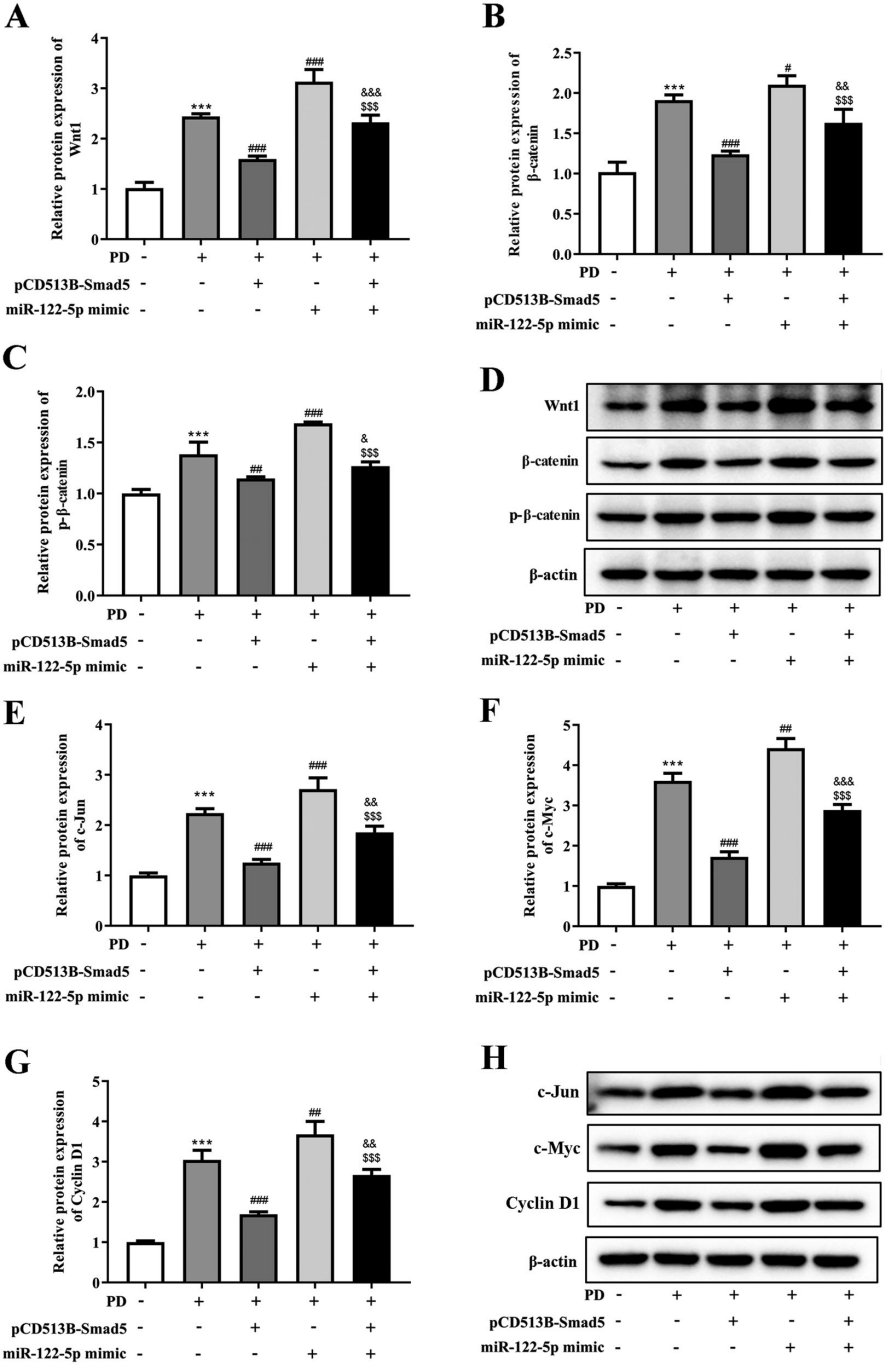
Overexpression of miR-122-5p enhanced PD-induced activation of canonical Wnt/β-catenin signaling pathway by targeting Smad5. Western blot showed the expression of Wnt1, β-catenin, p-β-catenin, c-Jun, c-Myc, and Cyclin D1 in peritoneum of different groups. ***p<.001 *versus* the control (Con) group. ^#^*p*<.05, ^##^*p*<.01, ^###^*p*<.001 *versus* the PD group. ^&^*p*<.05, ^&&^*p*<.01, ^&&&^*p*<.001 *versus* pCD513B-Smad5 treatment group. ^$$$^*p*<.001 *versus* miR-122-5p mimic treatment group. *n* = 8 (Con group), *n* = 7 (PD group), *n* = 6 (PD + pCD513B-Smad5 group), *n* = 6 (PD + miR-122-5p mimic group), and *n* = 6 (PD + pCD513B-Smad5 + miR-122-5p mimic group).

**Figure 8.**
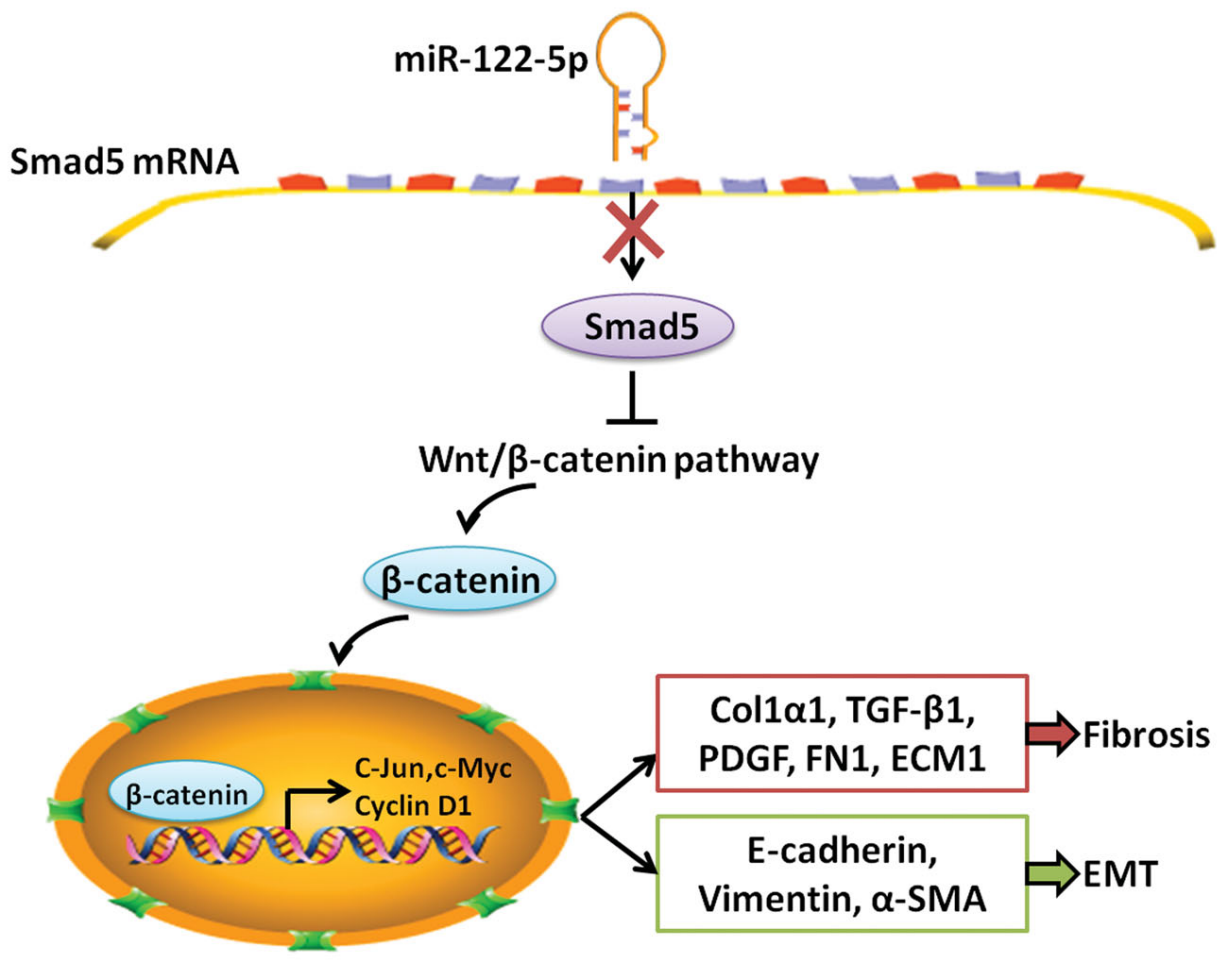
The mechanistic scheme of miR-122-5p in peritoneal fibrosis. As a negative transcriptional regulator of Smad5, miR-122-5p overexpression led to decreased expression of Smad5 in peritoneum. The decreased Smad5 promoted canonical Wnt/β-catenin signaling. As a result, peritoneal fibrosis and epithelial–mesenchymal transition (EMT) were accelerated.

Finally, we explored potential mechanisms whereby miR-122-5p/Smad5 mediates PF. Western blot and RT-qPCR analysis revealed that overexpression of miR-122-5p in PD rat was associated with a marked activation of Wnt/β-catenin signaling in the peritoneal tissue (Figure 7(A–D)). Meanwhile, miR-122-5p mimic promoted the expression of target genes c-Jun, c-Myc, and Cyclin D1 in peritoneum (Figure 7(E–H)). In contrast, gene transfer with pCD513B-Smad5 before the established PD resulted in a significant inhibition of canonical Wnt/β-catenin pathway induced by miR-122-5p mimic (Figures 7 and 8).

## 4. Discussion

Long-term exposure of the peritoneum to non-physiological PD solution causes peritoneal mesothelial cells shedding, the accumulation of ECM, and the formation of PF ultimately [17]. The maintenance of the osmotic pressure gradient across the peritoneum in PD plays a crucial role in ultrafiltration. At present, glucose dialysate is still used to form the osmotic pressure gradient [18]. In our study, we found that the degree of PF was most pronounced in PD solution with high glucose concentration, compared with those with medium and low concentrations, including thicker peritoneal membrane, infiltration of mononuclear cells, and increases of fibrosis marker proteins.

Accumulating evidence has shown that miRNA plays a key role in regulating the progression of PD-related PF [12,19]. In this study, we found that miR-122-5p expression was upregulated in peritoneal mesothelial cells and vascular endothelial cells of PD rat model with PF. Consistent with the previous finding that serum miR-122-5p level were elevated in hepatic fibrosis [20]. On the contrary, the miR-122-5p expression was declined in skeletal muscle fibrosis and overexpression of miR-122-5p was retarded fibrotic process. It was proven that TGF-β1 was a profibrotic cytokine that plays a pivotal role in fibrosis [21]. PDGF, a potent chemotactic factor for fibroblasts, was reported to induce the generation and sedimentation of collagen, which was significant in fibrosis development [22]. This study suggested that miR-122-5p overexpression promoted TGF-β1 and PDGF expression in PD rat. Furthermore, we have found that miR-122-5p overexpression enhanced the progression of EMT. Previous studies found that the establishment of PF was associated with the EMT of the peritoneal mesothelial cells [23,24]. EMT leads to the transformation of peritoneal mesothelial cells into fibroblasts, producing a large amount of ECM, which is a key process of peritoneal function degradation [25]. Meanwhile, EMT weakens the interaction between cells through regulating intercellular cadherin and β-catenin, and reshapes the cell scaffold through regulating the expression of α-SMA [26].

The study also found that PF leads to decreased Smad5 expression. Interestingly, using the dual-luciferase reporter system, we found that Smad5 was a target gene of miR-122-5p. Meanwhile, that miR-122-5p overexpression suppressed the expression of Smad5, confirming the regulation relationship between miR-122-5p and Smad5. In recent years, the role of Smad5 in regulating organ fibrosis has been established. Ablation of Smad5 caused liver injury and fibrosis in mice [27]. In subretinal fibrosis, overexpression of αB-crystallin enhanced nuclear translocation and accumulation of Smad4 and Smad5, which is related with EMT of retinal pigment epithelium cells [28]. In this study, we also found that Smad5 overexpression inhibited PF progression and EMT. Moreover, Smad5 overexpression blocked the aggravated PF and EMT progression induced by miR-122-5p overexpression.

Wnt protein is a type of secreted lipidated glycoprotein rich in cysteine, which is involved in cell differentiation, proliferation, and migration [29]. The Wnt signaling pathway, an essential component in early development, helps control cell differentiation and polarity through at least three distinct pathways [30]. The canonical Wnt pathway acting through activation of β-catenin is the best characterized [31,32]. In recent years, the role of Wnt/β-catenin signaling pathway in regulating EMT during organ fibrosis has been established [33]. The inhibition of Wnt/β-catenin signaling pathway limited collagen abundance and suppressed the progression of renal fibrosis [34]. Particularly, the activation of Wnt/β-catenin signaling pathway plays a key role in PF. Clinically, the higher expressions of Wnt1, Wnt5a, β-catenin, and LEF1 were observed in patients with more than 1-year PD compared with patients who just started with PD [35]. It has also been reported that Wnt/β-catenin signaling pathway induces EMT in human peritoneal mesothelial cells [35,36]. Our study proved that Wnt/β-catenin signaling pathway was activated in peritoneum of PD rat. MiRNA-122-5p mimic significantly strengthened the expression of Wnt1 and β-catenin and the expression of target genes c-Jun, c-Myc, and Cyclin D1, which could be reversed by Smad5 overexpression, suggesting that miRNA-122-5p/Smad5 axis maybe induce PF through regulating the Wnt/β-catenin signaling pathway.

In conclusion, this study found that miR-122-5p targeted and negatively regulated Smad5, thereby promoting the development of PF and EMT progression by mediating Wnt/β-catenin signaling pathway. These findings suggest that miR-122-5p may be a promising marker for the prevention or treatment of PF.

## Abbreviations

PF: Peritoneal fibrosis
PD: peritoneal dialysis
EMT: epithelial–mesenchymal transition
SD: Sprague-Dawley
TGF: Transforming growth factor
PDGF: platelet-derived growth factor
FN1: Fibronectin 1
ESRD: end-stage renal disease
3'-UTRs: 3'-untranslated regions
H&E: Hematoxylin and eosin
RT-qPCR: Real-time fluorescence quantitative polymerase chain reaction
